# Comparative investigation of the growth-poverty-inequality trilemma in Sub-Saharan Africa and Latin American and Caribbean Countries

**DOI:** 10.1016/j.heliyon.2020.e05631

**Published:** 2020-12-04

**Authors:** Bosede Ngozi Adeleye, Obindah Gershon, Adeyemi Ogundipe, Oluwarotimi Owolabi, Ifeoluwa Ogunrinola, Oluwasogo Adediran

**Affiliations:** a Department of Economics and Development Studies, Covenant University, Nigeria; b Centre for Economic Policy and Development Research (CEPDeR), Covenant University, Nigeria; c Regional Centre of Expertise (RCE), Ogun, Nigeria

**Keywords:** Economic growth, Poverty, Inequality, Sub-Saharan Africa, Latin America and the Caribbean, Economic development, Macroeconomics

## Abstract

To “*end poverty in all its forms everywhere*” and “*reduce inequality within and among countries*”, this study aligns with the 2030 Sustainable Development Goals 1 and 10. It uniquely contributes to the growth-poverty-inequality discourse by using per capita consumption expenditure growth (poverty), Gini index (inequality) and GDP growth (economic growth). It is a comparative analysis of 58 Sub-Saharan Africa (SSA) and Latin American (LAC) countries (from 2000 to 2015) to determine whether economic growth reduces the incidence of poverty and if its interaction with income inequality enhances or alters its impact on poverty. Consistent findings from a multi-analytical approach using pooled ordinary least squares, fixed effects and system GMM reveal that: (1) economic growth exhibit poverty-reduction properties; (2) the growth rate of inequality intensifies poverty, (3) inequality aggravates the impact of growth on poverty, and (4) the growth-poverty-inequality trilemma differs across income groups and regional samples. Furthermore, this study submits that the interaction of income inequality dampens the positive impact of economic growth on the incidence of poverty and supports the argument that the extent of inequality lessens the effect of inclusiveness. Hence, income inequality is a crucial determinant of poverty. Policy implications are discussed.

## Teaser

Economic Growth; Poverty; Inequality; Sub-Saharan Africa; Latin America and the Caribbean; Inequality; Poverty; Economic Development; Economic Growth; Macroeconomics

## 1 Introduction

This paper questions the growth-poverty-inequality trilemma by presenting empirical discoveries which fill a lacuna in the literature. This investigation takes a new perspective and highlights findings on whether economic growth reduces the incidence of poverty and if its interaction with income inequality improves or dims its impact. Conclusions reveal, *inter alia*, that though growth exerts poverty-reducing tendencies the interaction with inequality yields negative outcomes sufficient to dampen the poverty-reducing impact of economic growth. In essence, income inequality is a crucial determinant of poverty level. These are significant contributions to the growth-poverty-inequality literature, which provides the justification for engaging in this study – especially, from a cross-regional perspective.

From the literature and amidst recent economic growth in the global south, poverty is prevalent in Africa and appears to be worsening by widening inequality across the globe (Fosu, 2018). Inequality manifests as income gap, gender inequality, inequality of opportunity, as well as, differences in standard of living - like health inequality and energy inequality (Brunori et al., 2019; Ramosa et al., 2020; and Gershon et al., 2019). Despite economic growth recorded in developing countries, poverty and especially inequality are observable within cities, across regions in such countries, as well as, across regions within continents and in the global south (Clementi and Molini, 2019). In this regard, the Sub-Saharan Africa (SSA) and Latin America and Caribbean (LAC) countries readily come to mind because they are mostly developing. Against this background, it becomes important to investigate the growth, poverty and inequality trilemma.

From a pro-poor growth paradigm, the study is a comparative analysis of the transformation of GDP growth into the reduction of poverty while controlling for inequality in both regions. The notion of GDP growth as a measure of economic growth does hide the nature of regional and country-specific differences/similarities in poverty and inequality (Fosu, 2017; Adeleye et al., 2017). The growth-poverty-inequality nexus can be scrutinized from global, national and microeconomic lenses (Thorbecke, 2013), but this study uniquely considers it from a cross-regional perspective. Focusing on such countries is pertinent because of their structural macroeconomic differences but similar political upheavals. Furthermore, many developing countries in SSA and LAC have abundant natural resources and exporters of primary raw materials (Fosu and Abbas, 2019). Therefore, it becomes vital to understand the relative nature of poverty and inequalities in these regions.

Income inequality tends to increase in emerging economies with economic growth, but the opposite obtains in the highly developed countries (Soava and Sterpu, 2020). Moreover, expressions of poverty and inequality, amidst GDP growth, are exacerbated by climate disruptions, global health pandemics (like COVD-19) and macroeconomic shocks. It is a timely study as countries strive towards achieving Sustainable Development Goals (especially SDGs 1 to 4). On the originality of this research, some studies (Bourguignon, 2003; Fosu, 2009; Kanbur, 2016; Marrero & Serv'en, 2018) examined the tripartite connection of growth, poverty and inequality in a variety of ways using different measures of poverty, we analyse similar scenarios, but due to several countries not having data on poverty rate, we use per capita consumption expenditure as the measure of poverty (Gore et al., 1994; Johnson, 2004; Pape and Mistiaen, 2018; Stoyanova and Tonkin, 2018). In addition, an all-encompassing growth-poverty-inequality nexus using both descriptive and econometric analysis will be evaluated.

To probe the discourse, a panel data of 58 countries (*N*) across LAC and SSA for 16 years (*T*), and five variables – per capita consumption expenditure, GDP growth rate, Gini index, education and unemployment - are analyzed from 2000 to 2015. The starting year of 2000 is chosen because data on per capita consumption expenditure (proxy for poverty) is available for most of the countries from mid-2000 while the cut-off year of 2015 is due to the fact that data on Gini index (proxy for income inequality) for most countries ends at 2015. Following Ho and Iyke (2018); Garza-Rodriguez (2018); Uddin et al. (2014), we use per capita consumption of the household as the proxy for poverty. Furthermore, improving on existing methods in the literature (Bourguignon, 2004; Fosu, 2017; Marrero & Serv'en, 2018), static and dynamic models are adopted to address the issues of growth, poverty and inequality. The outcome of this study offers a new explanation for interpreting inequality in both regions. Furthermore, it presents new, and potential policy options for consideration by governments in the countries investigated.

To achieve the objective of the study which is to investigate whether economic growth reduces the incidence of poverty and if its interaction with income inequality improves or dims its impact on poverty, a multi-dimensional approach is adopted with estimations initially performed on (i) full sample of 58 countries, (ii) income groups and (iii) the region-specific sub-samples. This methodology makes the study holistic to ensuring a critical examination of its core argument. The rest of the paper is structured as follows: section 2 discusses the literature; section 3 gives some stylized facts; section 4 outlines the data and empirical model; section 5 discusses the results, and section 6 concludes.

## 2 Brief literature review

The last few decades have witnessed the economic prominence of developing and emerging economies. This is evident where many of those countries recorded considerably high growth rates above their developed counterparts. However, many of these economies have not experienced significant poverty reduction, which is traceable to high income inequality and persistent economic fluctuations as witnessed in many African countries in the 1980s and 1990s. In explaining how the significant growth experience of Sub-Saharan Africa and Latin America and Caribbean Countries can assist in improving human development and alleviates poverty, it is imperative to understand the importance of income equality in the growth-poverty nexus in the literature. For instance, Bourguignon (2003); Fosu (2009, 2015), Ho and Iyke (2018), Garza-Rodriguez (2018) and World Bank (2006a, 2006b), provide evidence that inequality aggravates poverty. The studies further suggest that the countries with the same level of economic growth may not likely attain a similar economic level of poverty reduction. Hence, attaining the United Nations Sustainable Development Goal (SGD) 1 (to end poverty) by developing and emerging economies may not be achieved in the same period.

In the same vein, the literature is inundated with different findings on the growth-poverty-inequality relationship, and without claiming to be exhaustive, some of them are highlighted. Škare and Družeta (2016) perform a synthesis of the literature in relation to poverty and economic growth while exploring possible inter-relationships between poverty and economic growth. Various theoretical approaches have been used to analyze poverty and growth in the literature such as those by Kuznets (1955), Bourguignon (2004), Iyke and Ho (2017) and Fosu (2011). Empirical studies reviewed indicate that analyses of growth and poverty have often been performed empirically with the use of survey data and large panel data sets. Further observation of the link between economic growth and poverty level across countries also reveal the presence of variations across regional blocks with regard to the effects of GDP growth on poverty reduction, thus suggesting that under different conditions, similar rates of growth can have very different effects on poverty. While according to economic literature, it tends to be popular that economic growth is of benefit to the poor, such growth may not be long-lasting unless the strategy of poverty reduction is founded on sustained economic growth. Hence, the methods used to measure poverty, absorptive capacity of the poor and the pace and pattern of economic growth are considerations when exploring the effect of economic growth on poverty.

According to Marrero and Serven (2018), both growth and poverty are related to income inequality. For instance, on the basis of estimating a reduced-form growth equation with the inclusion of measures of poverty, inequality and standard growth determinants, the relationship between poverty, income inequality and economic growth is consistent with the analytical framework of a learning-by-doing and knowledge spill-over model. In such a model, individuals given that their initial endowments are below a minimum consumption level are unable to save and invest. Thus, the study finds that subject to inequality, growth and poverty are negatively correlated, and in contrast subject to poverty, growth and income inequality can be positive or negative depending on the empirical specification and econometric approach. Such findings are further explained by the nature of data characterized by sample observations featuring high poverty level.

The arguments of Marrero and Serv´en (2018) imply that policies to improve growth, income inequality, and poverty must take into account the inter-relationships between each of poverty, income inequality and economic growth. Consistent with this argument is the triangular relationship of poverty with both income inequality and economic growth as highlighted by Hassan et al. (2015). Related to the aforementioned triangle of poverty, income inequality and poverty, is environmental degradation as argued by Hassan et al. (2015) who find an inverted u-relationship between economic growth and environmental degradation as measured by carbon dioxide emissions as well as a relationship amongst economic growth, income inequality and poverty in Pakistan from 1980 to 2011. Thus the environmental Kuznets curve hypothesis is of relevance for policy in Pakistan. Furthermore, preservation of the environment in Pakistan through, for example, economic transport system regulations is essential regardless of the level of income of the country.

Hence, this study presents regional and comparative country evidence on economic growth, inequality and poverty reduction in Sub-Saharan Africa (SSA) and Latin America and Caribbean (LAC) countries. It further contributes to the debate on the growth-poverty-inequality nexus in developing and emerging markets. The study achieves this by engaging a comparative investigation of the interaction of growth, poverty and income inequality from a sample of 58 SSA and LAC countries from 2000 to 2015. It will further assess the extent to which the recent consistent economic growth of developing and emerging economies could translate into poverty reduction. It is worthy of note that none of the previous studies had attempted to achieve this. Therefore, this study provides country and regional estimates of the relative contributions of inequality and income to the behaviour of poverty in the selected economies, and situate the trend on poverty reduction in the era of better economic growth of the developing economies. By this evidence, this study explains how average income growth and income distribution are important factors responsible for poverty reduction. Furthermore, while the evidence of average income growth reduces poverty globally as reported by Dollar and Kraay (2002) and Fosu (2017), this present study would suggest the relevance of income distribution in poverty reduction with a view to inform policy debate for focused research as well as contributions towards attaining SDG1 in developing and emerging economies.

## 3 Some stylized facts

This section delves into some comparative statistics using the core variables of the study - per capita expenditure (a proxy for poverty), GDP growth and Gini index - to show the trend analysis over time in these regions. The descriptive analysis gives in-depth details on the pattern of growth, poverty and income inequality for LAC and SSA countries (see Appendix for list) from 1980 to2018^2^.

### 3.1 Per capita consumption expenditure (PCE) growth

**Figure 1:**
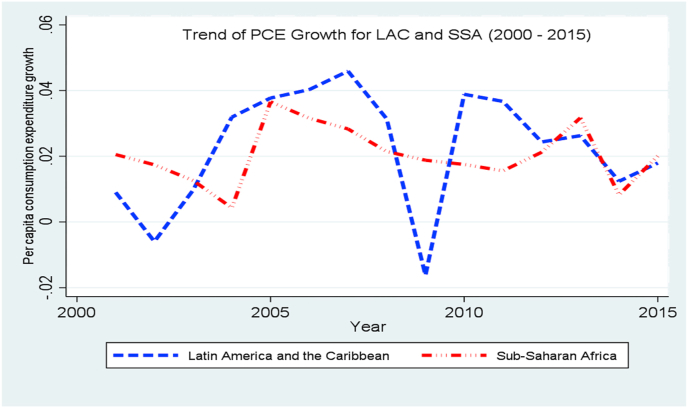
Trend of PCE growth for LAC and SSA Countries (2000–2015). Source: Authors.

From Figure 1, the pattern of per capita consumption expenditure (PCE) growth shows that with the exception of years 2001–2003, 2009, 2013 and 2015 where the PCE growth rate of SSA countries exceeded those of LAC countries by 0.002%–0.035%, the PCE growth of LAC countries is consistently higher. In level form (not shown), the per capita expenditure of LAC countries trends far above those of SSA countries which implies that Latin American households spend more on consumption than their African counterparts. Since poverty is measured through consumption deprivation (Gore et al., 1994; Stoyanova and Tonkin, 2018) the graphical illustration gives the indication that the poverty level in LAC is lower than those of SSA countries.

### 3.2 GDP growth

**Figure 2:**
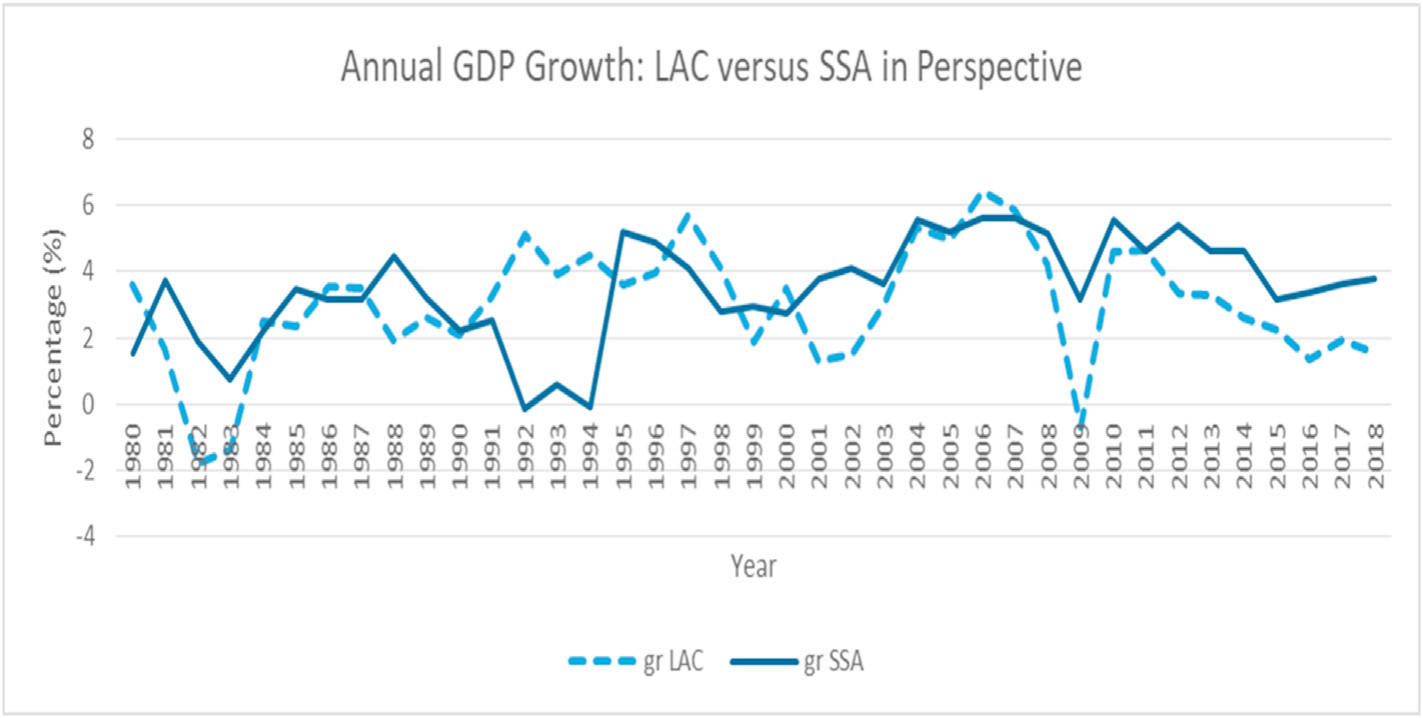
Trend of GDP growth for LAC and SSA countries (1980–2018). Source: Authors.

From Figure 2, within a thirty-nine-year horizon, yearly aggregate income growth estimates for LAC and SSA countries are plotted and can be viewed to be episodic, with some periods experiencing downward trends and others, upward trends. In the earlier periods, between 1981 and 1990, higher annual GDP growth is observed in the SSA region, with the highest record reaching a marked 4.4% in 1988 while LAC countries record an averaged 1.9% in the same year. Within the next ten years, aggregate income growth in the LAC region surged, relatively, above those of the SSA countries, as African economies struggle to keep above the positive band of income growth. Within that period, particularly in 1997, LAC's GDP growth had reached an all-time high of approximately 5.8%. Perhaps, the most successful periods for the two regions occurred between the early 2000s, where a decent recovery, occasioned by the smoothest rise of all times, is seen in both regions. Between 2001and 2007 for instance, LAC's recorded a gradual GDP growth: approximately 1.3%, 1.5%, 3.0%, 3.5%, 5.0%, 6.4% and 5.9% respectively. For SSA countries within the same period, annual growth in aggregate income recorded: approximately 3.8%, 4.1%, 3.6%, 5.5%, 5.2%, 5.6% and 5.6% for the respective years as well.

By 2008, the world had been hit by a global shock; the housing market bubbles created in 2006 had caused massive bank failures, and this brought with it, turns of investment downturns. This failure created a contagion among world economies and led to a total collapse of most markets globally. A significant decline in customer wealth and indeed, global economies prosperity valued in several trillions in US dollars was recorded and adjudged the worst economic meltdown since the Great Depression. In 2008, GDP growth in the SSA and LAC economies was observed to nosedive, with LAC countries taking the greater fall. For instance, in 2007, annual GDP growth for LAC was approximately 5.9%, and then, dropped to 4.2% the following year, 2008. By 2009, GDP growth in the region had crossed the negative bound, dipping as low as -0.7%. A recovery is observed from the year 2010 as world economies made efforts to revive from the 2008/2009 global collapse, which halted economic activities. A 4.6% growth rate was recorded in 2010, and this figure remained the same in the following year. About the same story can be told for the Sub-Saharan African region during the same period, although, the experience was less toxic. In 2007, GDP growth figure was 5.6%, then 5.2% in the following year; a significant drop was experienced in 2009 as GDP growth fell to 3.2%, while the region's recovery to a growth rate of 5.6% in 2010 is observed. Beyond this period, income growth per year for these two regions has fluctuated mildly.

### 3.3 Gini Index

**Figure 3:**
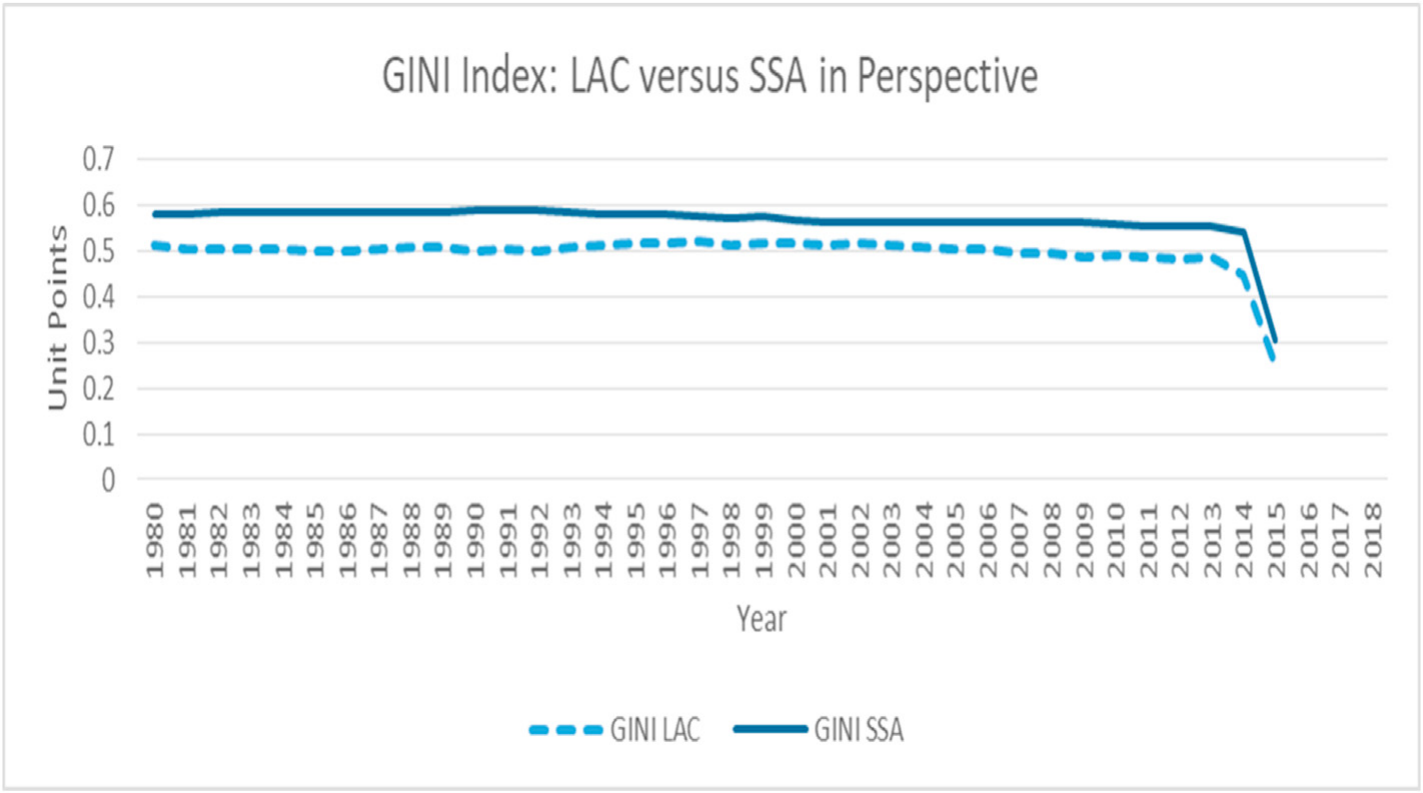
Trend of Gini index for LAC and SSA countries (1980–2015). Source: Authors.

The Gini index, a well-accepted measure of inequality, calibrates the percentage of income distribution among individuals in a country relative to the entire population. Higher Gini index figures portray higher levels of inequality and vice versa. SSA countries have, during the observed period, shown a higher level of inequality relative to the LAC countries. While it appears that the average Gini index value for SSA countries are sluggishly declining, those of the LAC countries appear to be comparably more constant over the years studied. Global efforts to reduce country-by-country inequality and terminate extreme poverty has remained the premier objective of the stainable Development Goals (SDG-1). These inequality and poverty reduction efforts have been helpful in some ways to morph some individuals out of the social and economic vulnerability net. For instance, the global poverty rate declined from 28 % in 1999 to 11 % in 2013 (Muhammad, 2020). Within the same time period, inequality in the LAC and SSA countries appear to sluggishly respond with a downward trend (see Figure 3). This, according to Muhammad (2020), means that the world's extremely poor population was 1.7 billion in 1999, and by 2013, there were just 767 million poor persons. By 2015, world poverty had dropped by an additional 1 % (Muhammad, 2020; World Bank, 2020). Figure 3 also reports a declining trend beyond 2012, reflecting Muhammad and World Bank's poverty report.

The possession of the larger proportion of total income by the few net-worth individuals in SSA countries may not come as a surprise, as increased cases of corruption, fiscal leakages and institutional short-circuiting have been a global concern for many years. Most African government officials are known to be opaque in dealing with public affairs, while they also create room for their allies in the private sector to further defraud their nations. Poverty incidence in these countries, therefore, continue to rise, as global efforts to combat this default remain a shadow chase. Furthermore, ensuring that only less than 3 % of the world's population live below $1.90 per day by 2030 may be a hard chase as the rate at which inequality and poverty declines seem sluggish (Muhammad, 2020). For instance, the data plot from Figure 2, Figure 3 shows that between 1980 and 2014, the average rate of decline in the Gini index for LAC and SSA countries are -1.83 % and -1.62 % per annum respectively. This realization corroborates the argument that significantly reducing inequality and keeping extreme poverty at bay by 2030 may be a hard catch.

## 4 Data and model

### 4.1 Data

The sample covers 58 selected LAC and SSA countries from 2000 to 2015. In line with existing literature, the study uses five variables: per capita consumption expenditure (proxy for the level of poverty), GDP growth rate (a measure of economic growth), Gini Index (a measure of inequality), secondary school enrollment and unemployment. The dependent variable is poverty; the main explanatory variables are economic growth and income inequality, while the control variables are secondary school enrollment and unemployment. Variables description and sources are listed in Table 1.

**Table 1:** Variables description and sources.

Variables	Description	Source
Per capita consumption expenditure growth	Proxy for poverty	PovcalNet
GDP growth rate (%)	Annual growth rate of GDP	World Bank (2019) WDI
GDP per capita (USD 2010)	GDP divided by the population	-do-
Secondary school enrolment rate	Ratio of children of official school age	-do-
Unemployment rate	Share of the labour force that is without work but available for and seeking employment.	-do-
Gini Index	Measure of income inequality	Lahoti et al. (2016)
Gini Index growth rate (%)	Growth rate of income inequality	Computed by Authors

### 4.2 Summary statistics and correlation analysis

The relative statistics of these indicators are shown in Table 2, while the correlation analysis is displayed in Table 3. Limiting discussions to the three variables of interest, per capita consumption expenditure, economic growth, and income inequality, the properties of the variables in Table 2 are elucidated using the full sample, regional and income groupings. The sample average for per capita consumption expenditure growth is 0.021% with Liberia (low income, SSA) having the lowest at -0.437% in 2004 and Nigeria (lower middle-income, SSA) shows the highest in 2001 with 0.44%. The standard deviation of 0.064 reveals a minimal dispersion from the sample means. Similarly, the sample average value for economic growth is 4.33% with a standard deviation of 4.132 evidencing wide dispersion from the sample mean. The country with the lowest growth rate of -36.39% in Central African Republic (low income, SSA) in the year 2013 while Chad (low income, SSA) shows the highest of 33.63% in 2004. Also, the country with the highest per capita income is Venezuela (Upper middle-income, LAC) with US$14,920.45 in 2008 while the lowest value of US$221.09 is recorded for Burundi (low income, SSA) in the year 2005. The standard deviation of 3500.73 reveals that the countries are hugely dispersed from the sample average of US$3,363.45.

**Table 2:** Summary statistics.

Groups	Statistics	PCEGR	GR	PC	GINI GR	GINI	SEC	UNEM
Full Sample	Mean	0.021	4.326	3363.448	-0.004	0.557	49.216	8.014
	Std. Dev.	0.064	4.132	3500.725	0.034	0.059	23.974	6.754
	Minimum	-0.437	-36.392	221.096	-0.261	0.390	3.280	0.299
	Maximum	0.441	33.629	14920.450	0.181	0.852	90.544	36.147
LAC	Mean	0.023	3.587	6239.194	-0.006	0.496	66.745	7.050
	Std. Dev.	0.037	3.458	3644.170	0.049	0.045	12.832	3.607
	Minimum	-0.166	-10.894	1294.221	-0.196	0.390	24.498	2.007
	Maximum	0.140	18.287	14920.450	0.181	0.595	88.966	20.520
SSA	Mean	0.020	4.720	1830.368	-0.002	0.589	29.847	8.528
	Std. Dev.	0.076	4.403	2227.053	0.022	0.034	17.683	7.892
	Minimum	-0.437	-36.392	221.096	-0.261	0.488	3.280	0.299
	Maximum	0.441	33.629	10160.340	0.104	0.852	90.544	36.147
High Income	Mean	0.031	4.009	9916.766	-0.007	0.488	76.717	8.238
	Std. Dev.	0.050	4.548	2484.673	0.020	0.043	9.420	3.777
	Minimum	-0.166	-10.894	5419.109	-0.059	0.408	58.315	2.300
	Maximum	0.096	11.984	14722.370	0.040	0.568	88.966	19.590
Upper Mid-Income	Mean	0.025	3.566	6524.096	-0.005	0.534	65.550	10.592
	Std. Dev.	0.038	3.297	2781.309	0.049	0.076	11.922	6.916
	Minimum	-0.122	-8.856	2554.225	-0.261	0.411	24.498	2.506
	Maximum	0.144	18.287	14920.450	0.157	0.852	90.544	33.473
Lower Mid-Income	Mean	0.025	4.543	1973.194	-0.004	0.561	44.797	10.521
	Std. Dev.	0.072	3.332	930.994	0.035	0.051	18.072	8.295
	Minimum	-0.331	-4.387	809.506	-0.187	0.390	9.154	2.007
	Maximum	0.441	18.869	4578.289	0.181	0.647	76.684	36.147
Low Income	Mean	0.013	4.832	627.767	-0.002	0.584	19.966	4.267
	Std. Dev.	0.077	4.970	322.355	0.015	0.022	9.804	3.285
	Minimum	-0.437	-36.392	221.096	-0.194	0.527	3.280	0.299
	Maximum	0.288	33.629	1869.553	0.104	0.788	46.580	16.935

Note: PCE = per capita consumption expenditure growth; GR = GDP growth rate; PC = GDP per capita (USD 2010); EDUC = secondary school enrolment; UNEM = unemployment rate (ILO); LAC = Latin America and the Caribbean; SSA = Sub-Saharan Africa.

**Table 3:** Correlation analysis.

Variables	PCEGR	GR	PC	GINI	GINIGR	EDUC	UNEM
PCEGR	1.000						
GR	0.542	1.000					
PC	0.058	-0.242	1.000				
GINI	-0.020	0.151	-0.608	1.000			
GINIGR	-0.065	-0.018	-0.008	0.178	1.000		
EDUC	0.013	-0.282	0.821	-0.584	0.027	1.000	
UNEM	0.044	-0.163	0.336	0.193	0.007	0.227	1.000

Note: PCE = per capita consumption expenditure growth; GR = GDP growth rate; PC = GDP per capita (USD 2010); EDUC = secondary school enrolment; UNEM = unemployment rate (ILO).

On inequality, the average growth rate of the Gini index is -0.004% with the lowest value of -0.261% from South Africa (upper middle-income, SSA) while the highest value of 0.181% is recorded for El-Salvador (lower middle-income, LAC) in 2008. The average Gini index is 0.557, and the standard deviation of 0.059 shows that the countries are clustered around the mean. The country with the highest inequality index of 0.852 is South Africa (upper middle-income, SSA) in 2010, while the lowest of 0.390 is from El-Salvador (lower middle-income, LAC) in 2008. Comparatively, poverty rate is lower in LAC (-0.166) than in SSA region (-0.437), economic growth is lower in LAC (18.29%) relative to SSA (33.63%), per capita income is highest in LAC (US$14,920.45) than in SSA (US$10,160.34), and Gini index is highest in SSA (0.852) relative to LAC (0.595). The correlation analysis shown in Table 3 reveals that economic growth, per capita income, education and unemployment exhibit positive association with per capita consumption growth while Gini index and its growth rate are negatively related to poverty rate. Connections among the regressors do not suggest the presence of multicollinearity as all the correlation coefficients are below 0.75. It is important to mention that the observed interactions are insufficient to conclude on the impact of the regressors on the outcome variable, hence the need to subject the relationships to rigorous empirical tests.

### 4.3 Empirical model

From the literature, the most significant poverty-reducing agent is economic growth (Dollar and Kraay, 2002; Fosu, 2009, 2015, 2017; Garza-Rodriguez, 2018; Iyke and Ho, 2017). However, African countries in particular are experiencing high growth rates without the corresponding poverty-reduction experience (Adeleye et al., 2017; Adeleye, 2020). This anomaly is due to the influence of inequality in the conversion of growth to poverty reduction, and the distributional effect of economic growth. Econometric modelling and *a priori* expectations for this study lean on the theoretical account of the growth-inequality-poverty triangle (Dhrifi, 2015), as was first used by Bourguignon (2004). The intervening relationship among the trio holds that income inequality worsens poverty incidence while growth forces down growing poverty rates. Also, income elasticity of poverty (expectedly) portrays a stronger influence than growth effects, indicating a stronger positive effect on poverty by inequality than growth. Highlighting this pivotal relation of growth and inequality on poverty reduction, this study follows but modifies Fosu (2017) by specifying a poverty model that includes an interaction of growth with initial inequality (Ravallion, 1997; Easterly, 2000). We also include two vital elements of pro-poor growth - education and unemployment – since they portray a contemporary strategic doctrine of progress in human capital development which may be altogether reflected in varied poverty levels of countries. While improvement in educational levels is expected to exert a southward pressure on poverty, the same will worsen if growing unemployment rates persist, *ceteris paribus*. The modified model is, therefore, expressed in the relation below:

[1] $$PCEGR_{it}=a_{0}+a_{1}GROWTH_{it}+a_{2}\text{ln}PC_{it}+a_{3}GINIGR_{it}+a_{4}\text{ln}GINI_{it}+a_{5}GROWTH*GINI_{it}+a_{6}\text{ln}EDUC_{it}+a_{7}\text{ln}UNEM_{it}+e_{it}$$

where *PCEGR* is the growth rate of per capita consumption expenditure (proxy for poverty rate), *GROWTH* is economic growth, *PC* is per capita income in natural logarithm, *GINIGR* is the growth rate of the GINI index, *GROWTH∗GINI* is the interaction of growth and level of inequality, *EDUC* is secondary school enrollment in natural logarithm, *UNEM* is unemployment rate in natural logarithm, $a_{j}$ (where j = 1, 2, …,7) are parameters to be estimated, *i****,*** countries, 1, 2……..*N; t,* time, 1, 2…..*T*, $e_{it}$ is the error term.

From Equation [1] which addresses the study objectives, $a_{1}>0$ to establish the growth-reducing effect on poverty. A positive coefficient implies an increase in per capita consumption expenditure which is poverty-reducing. Similarly, $a_{2}>0$ an increase in per capita income should increase per consumption expenditure which is also poverty-reducing. In contrast, $a_{3},a_{4}<0$ to elucidate that both the growth and level of inequality will reduce per capita consumption expenditure and worsen poverty rate. $a_{5}$ is expected to be negative for a higher level of initial inequality would decrease the rate at which growth acceleration is transformed into poverty reduction. $a_{6}>0$ as education increases economic opportunities and hence increases per capita consumption expenditure which is poverty-reducing. $a_{7}<0$ implies that unemployment reduces access to basic amenities hence, reduces per capita consumption expenditure which is poverty-enhancing.

### 4.4 Estimation techniques

The study uses static and dynamic models to systematically achieve the research objectives. These estimation approaches are suitable and have been used by similar studies (Bourguignon, 2004; Fosu, 2017; Marrero & Serv'en, 2018; Iyke, 2017) in addition to using a short panel data of 58 countries (*N*) across 16 years (*T*), hence, *N > T*. Similarly, the adoption of these techniques serves as robustness for one another in order to observe the consistency of the relation among the variables of interest. The static models are the pooled ordinary least squares (*POLS*) which do not allow for heterogeneities across the panels, and the fixed effects (*FE*) model which recognizes panel heterogeneities while the dynamic model is the systems generalized method of moments (*sys-GMM*). The *sys-GMM* estimator is designed for short panel analysis and has the following assumptions about the data-generating process which includes the fact that the process may be dynamic, with current realizations of the dependent variable influenced by past realizations in addition to the fact that the regressors are not strictly exogenous and may be correlated with past and possibly current realizations of the error term. To further extend the discourse, the study adopts the methodological approach of Adeleye et al. (2020), Adeleye and Jamal (2020) and Adeleye and Eboagu (2019) to analyse the data along income groups delineations - high income, upper middle-income, lower middle-income, and low income – and regions to observe if the impact of the regressors on the outcome variable differs significantly across the sub-samples. For comparative analysis, the income groups analyses are undertaken using the *POLS* technique^3^ only.

## 5 Estimations and discussions

The study offers insights into the empirical linkage of growth, poverty and inequality in SSA and LAC economies. The presentation of empirical discoveries which fill essential gaps in the growth-poverty-inequality literature showcases findings on whether economic growth individually reduces the incidence of poverty and if its interaction with income inequality enhances or reduces poverty. Estimations begin with the income group analysis using the *POLS* technique as shown in Table 4, followed by regional analysis with the *FE* and *Sys-GMM* techniques displayed in Table 5. These results are compressed into composite tables to avoid proliferation. In particular, Table 5 shows the “main” and “robustness” results. Interpretations of Tables 4 and 5 are taken in turns.

**Table 4:** Full sample and income group analysis (dep. Var: PCEGR).

Variables	Full Sample	HI	UMI	LMI	LI
*Constant*	-0.0149	-0.1904	-0.0818∗∗	0.0425	0.2085
	(-0.52)	(-1.18)	(-2.05)	(0.51)	(0.68)
*GROWTH*	0.0249∗∗∗	0.0246∗∗	0.0114	0.0381∗∗	0.1062
	(3.76)	(2.09)	(1.60)	(2.52)	(1.19)
*PC, log*	0.0043	0.0217	0.0155∗∗	-0.0067	0.0069
	(1.05)	(1.07)	(2.19)	(-0.76)	(0.39)
*GINI, log*	0.0490	0.0457	0.0346	0.0759	0.3955
	(1.57)	(0.83)	(0.98)	(1.39)	(0.85)
*GINI Growth Rate*	-0.0529	-0.5575∗∗∗	-0.0464	-0.1084∗	0.0630
	(-1.38)	(-2.83)	(-0.93)	(-1.83)	(0.44)
*GROWTH∗GINI*	-0.0338∗∗	-0.0314	-0.0061	-0.0592∗∗	-0.1759
	(-2.43)	(-1.23)	(-0.42)	(-2.14)	(-1.14)
*EDUC, log*	-0.0008	0.0060	-0.0069	0.0081	-0.0170
	(-0.08)	(0.15)	(-0.67)	(0.75)	(-0.85)
*UNEM, log*	0.0054∗	-0.0055	-0.0035	0.0117∗	0.0095
	(1.77)	(-0.58)	(-0.70)	(1.72)	(1.60)
Time Dummies	No	No	No	No	No
No. of Obs.	360	39	139	96	86
No. of Countries	58	4	17	15	22
R-Squared	0.364	0.849	0.645	0.170	0.148
F Statistic	28.558∗∗∗	41.052∗∗∗	30.010∗∗∗	2.977∗∗∗	1.278

Note: ∗∗∗, ∗∗, ∗ represent statistical significance at the 1%, 5%, and 10% levels, respectively; PCEGR = per capita consumption expenditure growth; HI = high income; UMI = upper middle-income; LMI = lower middle-income; LI = lower income; t-statistics in ( ).

**Table 5:** Results.

Regions	Combined Result (SSA & LAC)		SSA Result		LAC Result	
Variables	FE (Main)	Sys-GMM (Robustness)	FE (Main)	sys-GMM (Robustness)	FE (Main)	sys-GMM (Robustness)
*PCE Growth Rate_1*		0.0926∗∗		-0.00520		0.0275
		(0.0370)		(0.0458)		(0.0175)
*GROWTH*	0.0287∗∗∗	0.0199∗∗∗	0.00792	0.0560∗	0.0155∗∗∗	0.0223∗∗∗
	(0.00549)	(0.00640)	(0.0494)	(0.0325)	(0.00582)	(0.00347)
*PC, log*	-0.0111	0.0510∗∗	-0.0520	0.0653∗∗∗	0.00378	0.00383
	(0.0266)	(0.0230)	(0.0586)	(0.0213)	(0.0246)	(0.00701)
*GINI Growth Rate*	-0.0332	-0.0256	0.0252	-0.195∗∗	-0.0301	-0.0585∗∗∗
	(0.0544)	(0.0370)	(0.195)	(0.0775)	(0.0404)	(0.0165)
*GINI, log*	0.0539	0.112	0.183	0.343∗∗	0.0146	0.111∗
	(0.0550)	(0.0774)	(0.290)	(0.165)	(0.0445)	(0.0643)
*GROWTH∗GINI*	-0.0424∗∗∗	-0.0236∗	-0.00869	-0.0871	-0.0142	-0.0291∗∗∗
	(0.0108)	(0.0137)	(0.0849)	(0.0545)	(0.0121)	(0.00736)
*EDUC, log*	-0.000474	-0.00144	0.000439	-0.00177∗∗∗	-0.000787∗	0.000413∗
	(0.000517)	(0.000945)	(0.00126)	(0.000540)	(0.000402)	(0.000216)
*UNEM, log*	-0.0148	-0.0144∗	-0.00587	-0.0244∗∗∗	-0.0189∗	-0.00198
	(0.00940)	(0.00806)	(0.0168)	(0.00758)	(0.0105)	(0.00446)
Constant	0.171	-0.235∗∗	0.462	-0.166	0.0565	0.0157
	(0.187)	(0.113)	(0.376)	(0.110)	(0.199)	(0.0524)
Time Effect	YES	YES	YES	YES	YES	YES
Observations	360	338	159	148	201	178
R-squared	0.356		0.043		0.702	
Number of Groups	48	48	28	28	20	20
*Hausman*	Yes		Yes		Yes	
*Adj. R*^*2*^			0.5957			
*F-test (prob)*	0.0000				0.0000	
*Wald test (prob)*		0.000		*0.000*		*0.000*
*AR (1)*		0.060		*0.048*		*0.017*
*AR (2)*		0.310		*0.210*		*0.110*
*Hansen J test*		0.758		*0.590*		*0.714*
*Sargan test*		0.912		*0.974*		*0.920*
*Instruments*		24		*24*		*24*

Note: FE = Fixed Effects; standard errors in parentheses; ∗∗∗p < 0.01, ∗∗p < 0.05, ∗p < 0.1.

### 5.1 Pooled OLS results

Results from Table 4 shows that growth is a statistically significant poverty-reducing variable. The coefficient on the full sample is 0.0249 which is significant at the 1% level suggesting that a percentage change in economic growth leads to an increase in per capita consumption expenditure by 2.5 per cent. Given that per capita consumption expenditure is a true indicator of poverty, this result explains that economic growth will enhance the consumption capacity of an individual to meet his needs which in this case is poverty-reducing. This outcome is supported by previous studies (Garza-Rodriguez, 2018; Alvaredo and Gasparini, 2015; Bourguignon, 2003; Hernández-Laos, 2010; Fosu, 2017). Similarly, economic growth is poverty-reducing for the high income (0.246) and upper middle-income (0.0381) samples - an indication that a percentage change in economic growth increases consumption expenditure by 2.5 and 3.8 percent, respectively. A clear pattern from the growth-poverty relation is that the coefficient is positive across all specifications though not statistically significant for lower middle-income and low income countries. The coefficient of per capita income, although positive in four out of five specifications, is only statistically significant at the 5% level for upper middle-income countries (0.0155). This suggests that a percentage change in per capita income results in boosting consumption expenditure by about 2 percent, on average, *ceteris paribus*. This outcome is expected as individuals are able to increase consumption when income rises. The growth rate of the Gini index shows poverty-enhancing tendencies with statistically significant negative coefficients on the high income (-0.557) and lower middle-income (-0.1084) countries. The interpretation is that unequal aggravation of the income distribution exacerbates poverty by 55.7 and 10.84 percent, respectively. This clearly shows the detrimental impact of inequality on poverty. This finding is similar to Fosu (2017), who found that poverty-inequality elasticity is higher than poverty-income elasticity. Similar to Osinubi and Olomola (2020), the Gini index exhibit no significant impact on poverty across all model specifications.

Another essential finding is the interaction of economic growth and Gini index, which shows to consistently aggravate poverty across all specifications. Given the negative coefficients of *GROWTH∗GINI* that is statistically significant at the 5% level for the full sample (-0.0338) and LMI countries (0.0592), these outcomes support Bourguignon ((2004) that poverty depends on economic growth and changes in the income distribution. That is, the poverty-reducing effect of economic growth is dimmed by widening inequality. Explicitly, for the full and LMI samples, the differential^4^ of -0.0089 (that is, 0.0249–0.0338) and -0.0211 (that is, 0.0381–0.0592) gives the *total* impact of *GROWTH* on per capita expenditure growth rate given *GINI* which shows that the negative interaction is sufficient to dampen the poverty-reducing impact of economic growth. This outcome is a significant incursion to the literature.

Education does not exert any statistical significance on poverty, while unemployment shows to be poverty-reducing for the full sample (0.0054) and lower middle-income countries (0.117). Both outcomes which seem implausible are significant at the 10% level. The most plausible argument for such outcomes can be linked to the payment of unemployment benefits which cushions poverty, in the short-term. On the model diagnostics, time dummies were not jointly significant in explaining variations in the outcome variable, hence their removal. The R-squared which ranges between 14.8% and 85% shows the variation in the outcome variable explained by the regressors. Lastly, the statistically significant F-statistic affirm that the regressors are jointly significant in explaining per capita consumption expenditure growth except for the low income countries sample.

### 5.2 Fixed effects and system GMM results

Table 5 presents the estimation results, showing the static (fixed effects) and dynamic (system GMM) panel analysis. It is not unlikely that growth can be explained by the extent of poverty in an economy. In such manner, when a lesser proportion of poor participate in the growth process, overall growth is hindered and inequality widens (Nallari and Griffith, 2011). In other words, creating pro-poor economic policies is dependent on appropriate measurement of poverty. A pre-estimation assessment reveals two explanatory variables, growth rate of GDP (*GROWTH*) and secondary education (*EDUC*) were not strictly exogenous and provides the need for robustness checks with the two-step system GMM with Windmeijer (2005) corrected cluster robust errors to overcome the problem of endogeneity in the model (Arellano & Bond 1991; Arellano & Bover 1995; Blundell & Bond 1998). According to Roodman (2009, 2014), the basic requirement for the adoption of GMM is met in the study. First cross sectional dimension, *N*, is moderately sized, hence, yielding a reliable Arellano-Bond autocorrelation test and cluster-robust standard errors.

Given that the underlying algorithm of the fixed effects (main analysis) and system GMM (robustness analysis) techniques differ considerably, results interpretations will emphasize (1) the consistency of the signs of the coefficients, and (2) their statistical significance, if any. Similar to Table 4, the *GROWTH* coefficient is positive across all model specifications and statistically significant at the 1% level except the SSA sample with fixed effects technique. These outcomes reveal that economic growth has poverty-reduction properties from between 0.0155 to 0.0560 percent which is consistent with similar studies (Alvaredo and Gasparini, 2015). The outcome supports the argument that GDP growth rate significantly influences the growth rate of per capita consumption expenditure causing a reduction in the burden of poverty (Garza-Rodriguez, 2018). Furthermore, the evidence is consistent with the assertions of emerging theories of growth and espoused in Mulok et al. (2012) 5. It is necessary to note that insomuch that the forgoing holds sway, the level or magnitude of poverty reduction would depend on the inclusiveness of the growth process. Using the GMM results, the magnitude is generally weak but relatively larger for SSA (5.6%), compared to LAC (2.2%) and the combined sample (2.0%). This implies that the rate of absolute poverty reduction induced by GDP growth should be faster in SSA.

The coefficient of *PC* is consistent across the GMM analysis with positive signs and statistically significant for the full (0.0510) and SSA (0.0653) samples at the 5% and 1% levels, respectively suggesting that increase in income increases per capita consumption expenditure which lowers poverty level (Fosu, 2017).

As expected, the negative coefficient of *GINI Growth Rate* is consistent in five of six models and informs that income inequality aggravates poverty as a result of the reduction in per capita consumption expenditure. Significantly, *GINI Growth Rate* exacerbates poverty level in LAC (-0.195) and SSA (-0.0585) countries at the 5% and 1% levels, respectively. This evidence portrays the increasing incidence of poverty in the economies in spite of impressive growth tracks and trade booms. For instance, in recent decades, SSA was adjudged a fast growing region yet home to the most economically deprived people (Adeleye et al., 2017). The evidence shows that a 100% increase in the *growth rate of GINI* offsets the welfare of vulnerable poor by 19.5% and 5.85% in SSA and LAC respectively, hence, leaving a net welfare of 14.8% and 5.25% in SSA and LAC respectively.

Remarkably, the result shows that rising income inequality considerably dwindles absolute poverty. As the economy expands, countries become internationally linked and attract investment into the space and livelihood of the poor. It first attracts more returns to owners of production factor and equally provides more jobs and opportunities (both farm and off-farm) which partly offsets the erstwhile unemployment (Ogundipe et al., 2019). The evidence is particularly obvious in SSA and LAC economies, though the effect seems larger for SSA. The rising relative poverty (*GINI*) raises welfare of the poor by 34.4% and 11.1% in SSA and LAC respectively. However, economic policy analysts need a keen attention on the growth rate of *GINI*, as a significant increase in the growth rate can spur a reversal of the initial inclusiveness attained. The evidence on the effect of the *GINI* further reinforces the relevance of equitable distribution of growth gains in stimulating general welfare. Across the samples, the positive coefficient of *GINI* suggests that increase in inequality increases per capita consumption expenditure (reducing the incidence of poverty) but only statistically significant for the SSA (0.343) and LAC (0.111) samples at the 5% and 10% levels, respectively.

More so, the interaction term *GROWTH∗GINI* (the extent of inclusiveness) exerts a negative influence on the growth rate of per capita consumption expenditure. Thus, it raises the incidence of poverty significantly for the full (-0.0424, -0.0236) and LAC (-0.0291) samples. This evidence significantly differs from the individual effect of *GROWTH* and *GINI* on the poverty indicator. The interaction term shows the intensification of poverty when compared with individual effects of *GINI* on poverty and GDP growth on the poverty indicator. This suggests that accounting for the role of income inequality weakens the expected positive impact of economic growth on general welfare and support the outcomes obtained in Table 4. Concisely, the differential of -0.0137 (that is, 0.0287–0.0424), -0.0037 (that is, 0.0199–0.0236) provides the *total* effect of *GROWTH* on per capita expenditure growth. This suggests that interaction with inequality dampens the positive impact of economic growth on the incidence of poverty. This is an important finding which supports the argument that income inequality exaggerates poverty irrespective of the positive impact of economic growth. That is, the extent of inequality reduces the effect of inclusiveness in the regions. The foregoing, hence reveals that inequality is a crucial determinant of the rising incidence of poverty in SSA and LAC economies, though, the effect was larger in SSA.

Education shows an inconsistent impact on poverty across the models. While it significantly aggravates poverty in SSA (-0.00177) at the 1% level, its impact in LAC is asymmetric (-0.000787, 0.000413) at the 10% level. Finally, as expected, the growth rate of per capita consumption expenditure responded inversely to a contemporaneous change in unemployment. As unemployment increases, the per capita consumption expenditure declines (raising poverty incidence). This significant impact is evident in for the full (-0.0144), SSA (-0.0244), and LAC (-0.0189) samples and implies that unemployment contributes significantly to poverty.

The post estimation diagnosis (presented in the lower panel of Table 5) shows that the parameters obtained are reliable, consistent and suitable for drawing inferences. On the choice of instruments used, Windmeijer (2005) recommends that mean biasedness of parameters are lowered when instruments counts are lowered. The assertion corroborates Roodman (2009) that proliferation of instruments can result in overestimation of parameters. In spite of the importance of instrument selection in addressing the simultaneity issue and its implication for GMM results (Iyke 2017, 2018) yet there is no theoretical consensus on the optimal number of instruments required when addressing the problem (León-González and Montolio, 2015).

## 6 Conclusion

To “*end poverty in all its forms everywhere*” and “*reduce inequality within and among countries*”, this study aligns with the 2030 Sustainable Development Goals 1 and 10 to examine the growth-poverty-inequality trilemma using per capita consumption expenditure as the indicator of poverty. It uniquely contributes to the debate on the growth-poverty-inequality nexus by engaging comparative analyses of the interaction of growth, poverty and income inequality from a sample of 58 Sub-Saharan Africa and Latin American and Caribbean countries from 2000 to 2015. Findings from both static and dynamic analyses across both full and sub-sample estimations reveal that (1) economic growth is a significant contributor to reducing the incidence of poverty, (2) the growth rate and income inequality intensifies poverty rate, (3) the interaction of economic growth and inequality aggravates poverty. The crucial finding is that the interaction of income inequality dampens the positive impact of economic growth on the incidence of poverty and supports the argument that inequality exaggerates poverty irrespective of the positive impact of economic growth. This is because of the distributional effect of economic growth. As such, the extent of inequality lessens the effect of inclusiveness – more so, during downturns. Consequently, it becomes important to consider introducing and/or reviewing policies that enhance the disposable incomes of poor households – especially in rural areas and urban slums. Perhaps, it may be more effective to combine economic growth target/strategies with poverty-reduction measures in SSA and LAC.

Additionally, the results obtained here are peculiar due to the cross-regional comparison of SSA & LAC countries - considering differences in tax systems, disposal incomes and household sizes within and across the regions. Besides taxation, assess to credit and entrepreneurship system are other factors that contribute to inequality in most developing and emerging economies. As such, from a policy perspective, it recommended that policymakers consider effective tax policies to sustainably reduce inequality – especially in the post-COVID-19 downturn. Furthermore, considering the youth in SSA and LAC, the demographic effects of monetary or credit access policies on economic growth and poverty reduction needs to be incorporated in policymaking. Meanwhile, it will be interesting to further analyse the nexus of economic growth, inequality and poverty reduction during recessions – as this will further enhance the results obtained here.

## Declarations

### Author contribution statement

B.N. Adeleye: Conceived and designed the experiments; Analyzed and interpreted the data; Contributed reagents, materials, analysis tools or data; Wrote the paper.

O. Gershon: Conceived and designed the experiments; Wrote the paper.

A. Ogundipe: Analyzed and interpreted the data; Wrote the paper.

O. Owolabi and O. Adediran: Contributed reagents; Wrote the paper.

I. Ogunrinola: Performed the experiments; Wrote the paper.

### Funding statement

This research did not receive any specific grant from funding agencies in the public, commercial, or not-for-profit sectors.

### Declaration of interests statement

The authors declare no conflict of interest.

### Additional information

No additional information is available for this paper.

**Appendix Table 1A:** List of Countries, Regions and Income Groups.

S/No.	Country	Region	Income Group
1	Angola	SSA	Lower Middle-Income
2	Argentina	LAC	High Income
3	Belize	LAC	Upper Middle-Income
4	Benin	SSA	Low Income
5	Bolivia	LAC	Lower Middle-Income
6	Botswana	SSA	Upper Middle-Income
7	Brazil	LAC	Upper Middle-Income
8	Burkina Faso	SSA	Low Income
9	Burundi	SSA	Low Income
10	Cabo Verde	SSA	Lower Middle-Income
11	Cameroon	SSA	Lower Middle-Income
12	Central African Republic	SSA	Low Income
13	Chad	SSA	Low Income
14	Chile	LAC	High Income
15	Colombia	LAC	Upper Middle-Income
16	Comoros	SSA	Low Income
17	Congo, Dem. Rep.	SSA	Low Income
18	Congo, Rep.	SSA	Lower Middle-Income
19	Costa Rica	LAC	Upper Middle-Income
20	Cote d'Ivoire	SSA	Lower Middle-Income
21	Dominican Republic	LAC	Upper Middle-Income
22	Ecuador	LAC	Upper Middle-Income
23	El Salvador	LAC	Lower Middle-Income
24	Eswatini	SSA	Lower Middle-Income
25	Gabon	SSA	Upper Middle-Income
26	Ghana	SSA	Lower Middle-Income
27	Guatemala	LAC	Upper Middle-Income
28	Guinea	SSA	Low Income
29	Guinea-Bissau	SSA	Low Income
30	Honduras	LAC	Lower Middle-Income
31	Jamaica	LAC	Upper Middle-Income
32	Kenya	SSA	Lower Middle-Income
33	Lesotho	SSA	Lower Middle-Income
34	Liberia	SSA	Low Income
35	Madagascar	SSA	Low Income
36	Malawi	SSA	Low Income
37	Mali	SSA	Low Income
38	Mauritania	SSA	Lower Middle-Income
39	Mauritius	SSA	Upper Middle-Income
40	Mexico	LAC	Upper Middle-Income
41	Mozambique	SSA	Low Income
42	Namibia	SSA	Upper Middle-Income
43	Nicaragua	LAC	Lower Middle-Income
44	Niger	SSA	Low Income
45	Nigeria	SSA	Lower Middle-Income
46	Panama	LAC	High Income
47	Paraguay	LAC	Upper Middle-Income
48	Peru	LAC	Upper Middle-Income
49	Rwanda	SSA	Low Income
50	Senegal	SSA	Low Income
51	Sierra Leone	SSA	Low Income
52	South Africa	SSA	Upper Middle-Income
53	Sudan	SSA	Low Income
54	Tanzania	SSA	Low Income
55	Togo	SSA	Low Income
56	Uganda	SSA	Low Income
57	Uruguay	LAC	High Income
58	Venezuela, RB	LAC	Upper Middle-Income

Source: Authors' Compilations

## Footnotes

2 The PCE growth trend analysis is restricted to years 2000–2015 due to many SSA countries having no data prior to year 2000.

3 The number of countries in each income group: High income (4), Upper middle-income (17), Lower middle-income (15), and Low income (22). Given that the data span is 16 years, only the sub-samples of Upper middle-income and Low income meet the criteria for performing FE and sys-GMM and because this will make comparative analysis impossible, this approach is dropped. Hence, only the POLS is used for comparative analysis.

4 The differential is obtained by deducting the coefficient of the interaction term from that of growth.

5 The study shows how rapid economic growth stimulate poverty reduction and remarkable social transformation in Malaysia.
